# The European university alliances—an examination of organizational potentials and perils

**DOI:** 10.1007/s10734-022-00951-4

**Published:** 2022-11-02

**Authors:** Peter Maassen, Bjørn Stensaker, Arianna Rosso

**Affiliations:** Department of Education, Faculty of Educational Sciences, University of Oslo, Oslo, Norway

**Keywords:** European integration, Higher education policy developments, European university alliances, Meta-organizations, Institutional theory

## Abstract

The European Union (EU) has repeatedly underlined the importance of higher education, research and innovation as drivers in the further development of Europe—economically, socially and culturally. One of the latest policy initiatives by the European Commission (EC) intended to promote this agenda is the European Universities Initiative (EUI) where alliances between universities across national borders are to identify new approaches for boosting European scientific cooperation. It might be argued that this development represents an attempt to find an organizational solution to the European policy ambitions in higher education, research and innovation. This article presents a framework for analysing European university alliances. Based on interviews with persons occupying key leadership and management roles in ten alliances, the article analyses the potential gains and perils alliances might face along four dimensions: their internal coordination, their ways of resolving conflicts, the commitment of member universities and the cultural characteristics of the alliances.

## Introduction

Over the last two decades, the European Commission (EC) has repeatedly underlined the crucial role of higher education, research and innovation in the further development of the European Union (EU). This growing attention for the knowledge sector is operationalized through several policy initiatives the EC has launched intended to boost European integration further—economically, socially and culturally (Chou & Gornitzka, 2014). One of these initiatives is the so-called knowledge triangle policy from the early 2000s that was driven by the idea that bringing higher education, research and innovation closer together across national borders would boost the global economic competitiveness of Europe as a region. However, analysis of the EU’s knowledge triangle policy implementation demonstrated the practical difficulties of aligning work of different EU DGs, and finding ways to coordinate policy initiatives related to core knowledge areas at European, national and regional level (Maassen & Stensaker, 2011: 766).

While finding ways to better coordinate the policy areas of higher education, research and innovation has remained high on the political agenda of the EC (2020a; 2020b), several coordination challenges have been identified in these areas of European policy making—driving the search for new policy solutions (Vukasovic et al., 2018).

With the recent EU’s “European Strategy for Higher Education” (European Commission, 2022), one could argue that a policy shift can be observed from an emphasis on policy coordination at EU level to an emphasis on organizational coordination at university level in the form of the development of university alliances. In essence, the strategy underlines the importance of cross-border cooperation between universities^1^ in Europe and how universities are crucial for the implementation of the political agenda of the EU (European Commission, 2022: 16). The effectiveness of the contributions of universities, it is argued, can be enhanced through cross-border cooperation where formal alliances of universities from various European countries would be an important building block.

This European strategy for universities is part of a higher education package, together with a proposal for a Council Recommendation on building bridges for effective European higher education cooperation. The strategy and recommendation aim to unlock the full potential of the higher education sector as the promoter of skills and knowledge and as an engine for innovation and solving societal challenges. Incentives for transformation of universities take a central place in the strategy, which is partly built on the first lessons learned from the EC’s programme to establish European university alliances, that is, the European Universities Initiative (EUI).

The EUI can be traced back to a speech by the French President Macron in 2017 (Jungblut et al., 2020). The ideas presented in this speech have been used to introduce the EUI programme resulting in the currently recognized 41 European university alliances, which include almost 300 universities in the European region. The Commission’s objective is to stimulate the further rollout of the EUI under the Erasmus + programme, while also linking this initiative with other EU instruments, such as Horizon Europe and the Digital Europe Programme (European Commission, 2020b). The EUI is novel in that it clearly signals an interest in an integrated use of the instruments at the disposal of the EC, at the same time as it demonstrates an interest in the potential of university alliances as an organizational solution to the challenges of effective policy coordination. While universities undoubtedly are key institutions for implementing policies in this area, the potential and capacity for alliances of universities to operate effectively across national borders is still rather unclear.

Hence, the purpose of this article is to examine the capacity of European university alliances as a tool for realizing the ambitions for creating the Europe of Knowledge. We address the following research questions in the article:

1. How can university alliances be theoretically conceptualized and analysed?
2. What are key potentials and perils of university alliances as an organizational form?

## The introduction of the EUI

The EUI is rooted in recent reforms in Europe aimed at enhancing the quality, relevance and effectiveness of the higher education sector (Maassen & Olsen, 2007). These reforms have mostly played out at the national level, and been heavily inspired by ideas from New Public Management (Shattock, 2014). While different policy mixes have been attached to these reforms, there are several common elements such as rationalized decision-making within universities, attempts to strengthen the strategic capabilities of universities and incentivize their links to society and the private sector (Capano et al., 2020; Gornitzka et al., 2017). Many reforms have been met with scepticism, not least because of the many unfounded assumptions underlying the reform agendas (Maassen & Olsen, 2007; Bleiklie et al., 2017), and unintended effects with respect to their implementation, including increased bureaucratization and added reporting (Bleiklie et al., 2017; Maassen, 2017).

The EUI is anchored in the policy idea that cross-border collaboration in European higher education should be moved to a new level, that is, from being based on time-limited projects to consisting of institutionalized long-term integration of academic activities, and in some cases, whole institutions. The latter would form the true “European Universities” of the future (Jungblut et al., 2020). This implies that the EUI wants to use traditionally rather self-standing EU programme tools, such as student mobility, joint degrees and mutual recognition of credit points and academic qualifications (Vukasovic, 2017), to enhance university integration.

The ambition of the EUI is to stimulate, “persistence, excellence and European values” through the selected alliances. These are further expected to offer student-centred curricula jointly delivered across an “inter-university” campus at all study levels, taking a “challenge-based approach” in which students, academics and external partners cooperate in cross-disciplinary teams to tackle the main issues facing Europe today, according to the Commission’s DG for Education and Culture (University World News, 2019).

In 2019, the EC selected the first 17 European university alliances. In 2020, the results of the second call for new alliances were published granting funding for 24 additional alliances. Through these two rounds of EUI calls, 279 European universities became part of a European alliance, being located in 32 countries spread throughout the continent. While each selected alliance receives a basic grant of € 5 million from the Erasmus + programme, additional EU funding for the alliances has been made available through earmarking Framework Programme (Horizon 2020 and Horizon Europe) resources adding research and innovation to the university alliance initiative.

However, it is important to underline that university alliances are not a unique European phenomenon (Sakamoto & Chapman, 2011). The establishment and increase in the number of alliances in higher education follow a more global trend where universities in numerous regions are joining up in various forms of formal inter-institutional collaboration (Stensaker, 2018; Vukasovic & Stensaker, 2018). What can be regarded as unique, though, is that the 41 selected European university alliances are the result of an external initiative, and not only driven by voluntary deliberations and decisions of the involved universities themselves. In addition, the external initiator is funding the selected alliances, with clear expectations on how the invested funds will be used, and to which outcomes they should lead.

The EC has expressed a number of ideas on what the selected alliances are expected to deliver, how to create a solid legal foundation for the new alliances and how to remove obstacles for their further integration (European Commission, 2020b; see also European Commission, 2022). While the legal foundation of these alliances is undoubtedly important, formal rules and regulations may have limited impact on the actual functioning of the alliances. In order to get a better understanding of whether these alliances will be able to accomplish what they have set out to do, there is a need for a more theoretically informed framework for analysing the key characteristics of European university alliances, their potential persistence and the extent to which they form a genuinely new element in the European knowledge landscape.

## A framework for analysing the promises and perils of university alliances

Institutional theories in general have proven very valid in explaining the shaping and continuity of organizational forms, including in higher education (Brunsson & Jacobsson, 2000; Djelic & Sahlin-Andersson, 2006; Greenwood et al., 2011; Maassen & Olsen, 2007). Within a sociological institutionalist framework, the history and identity of organizations (Selznick, 1957) along with environmental expectations and pressures (Scott, 2014) are identified as key dimensions to understand (converging) organizational adaptation and change. Such institutionalist frameworks have received criticism though, pointing to their inability to explain divergent change processes (Greenwood et al., 2011; Scott, 2014). Thus, over time, sociological institutionalist frameworks have increasingly taken on board insights from other theories including economic, instrumental and network approaches (Owen-Smith & Powell, 2008; Stark, 2009; Vedres & Stark, 2010).

When analysing European university alliances as an organizational form, we can draw on a range of theoretical insights including both institutional and instrumental dimensions. Universities have for long been recognized as organizations characterized by formal structures and rules, as well as informal rules and norms for organizing academic and cultural relations (Maassen & Olsen, 2007). University alliances can be expected to be carriers of much of the same cultural heritage, norms and values as their members, underlining an important cultural side of building an alliance. University alliances also represent the formation of a new organizational form having formal structures and decision-making bodies, making it important to bring in more instrumental perspectives when analysing the EUI. The meta-organization perspective opens for such an inclusive theoretical scope (Ahrne & Brunsson, 2005). Hence, the theoretical approach used in this article is to analyse European university alliances as “meta-organizations”.

### University alliances as multi-dimensional meta-organizations

Meta-organizations are special kinds of organizations characterized by the fact that other organizations, and not individuals, account for the membership (Ahrne & Brunsson, 2008). From an institutionalist perspective, it is assumed that such meta-organizational entities are drivers of ideas, interests and identities that make up the ingredients of institutional environments (Scott, 2014: 125). From this perspective, university alliances can be seen as organizations “taking over” their own environment in that they turn part of their environment into an organizational form (Ahrne & Brunsson, 2005: 447). In this respect, European university alliances are mechanisms linking up macro and micro levels in the European higher education area, representing a particularly interesting form of instrumental agency that can foster transformations in higher education.

However, while member universities in an alliance will have specific characteristics in terms of status, historical legacy, research output or financial situation, these characteristics may not automatically be transferred to the alliance they are member of. Meta-organizations, such as university alliances, can be considered potentially weakly integrated organizations in that members are expected to be equal, implying that no member is above another in hierarchical terms and consensus may be needed for agreeing on important decisions (Ahrne & Brunsson, 2008; Torfing, 2012).

Therefore, it should not be taken for granted that university alliances are fast-moving entities and that joint decisions are always embedded effectively in the individual member universities. Overall, the development of alliances and the actions taken by their members are often co-constitutive—they set the conditions of possibility for each other (Owen-Smith & Powell, 2008: 618). University alliances may incorporate both interesting dynamics and more predictable incrementalism, not least due to the legal status of individual members and lack of formal discretion these might have due to national legislation (Shattock, 2014).

It can be argued that alliances have the potential to form new institutional logics within the field of higher education. Alliances can be seen as organizational constructs where new knowledge is developed through specific practices and initiatives within the network they constitute (Vedres & Stark, 2010: 1184). What kind of “logics” dominates the different European university alliances might differ though, not least due to the complex reform trajectories of European countries (Bleiklie et al., 2017; Capano et al., 2020). Given that the alliances are made up of universities from countries with significant varieties in their political order (Fukuyama, 2015), one cannot merely assume that each alliance will develop along a similar trajectory as predicted in an institutionalist perspective. The establishment of university alliances has previously also been explained with reference to a resource-based view of organizations. University alliances are, from this view, created because organizations believe that they serve their economic interests in various ways (Beerkens, 2004). Furthermore, Inkpen and Tsang (2007) and Stensaker (2018) have argued that alliance formation rationales also include risk reduction and organizational learning.

Hence, it is possible to identify both institutional and instrumental arguments of potential validity for interpreting the formation and effects of university alliances. For example, although meta-organizations can be considered weakly integrated organizations, they can still be important agenda setters that instigate new standards and practices. In addition, they exert much external influence and can build legitimacy for their members (Ahrne & Brunsson, 2008; Torfing, 2012). Alliances could also represent innovation in organizational structures (Lounsbury & Crumley, 2007), or as a form of search process identifying interesting (economic) opportunities in the environment (Stark, 2009: 165). According to Stark (2009: 19), alliances can be described as heterarchies where a number of interdependencies exist, characterized by complex collaboration and most likely competing principles of performance and worth. The implication is that there can be internal dynamics within alliances shaping developments, and that the governance and internal organizing of alliances are highly important mechanisms for achieving the objectives set for the alliances.

To take advantage of the many possible opportunities of alliances is still challenging as intra-alliance collaborations—especially between similar types of organizations—also imply a form of internal competition (Inkpen & Tsang, 2007: 493). Network theorists have argued that the latter may be a key explanation for the fact that organizational alliances often can be characterized as unstable, ineffective and with quite poor performance (Muthusamy & White, 2005: 415). The risk of one partner behaving opportunistically exploiting the knowledge and assets of another is a potential danger in such collaborations. To make matters more complicated, organizations are usually embedded in a broader web of formal and informal networks and links, making it a challenging task to assess and identify what collaborations should be prioritized and which partners should be engaged with. Members of European university alliances can be argued to fit the latter description rather well as they also may be involved in a number of other external collaborations, including being members of other inter-university associations, coalitions and networks (Vukasovic & Stensaker, 2018).

For organizations that are part of strategic alliances, establishing trust within the group is often considered a key factor in explaining why partners share information, commit themselves and engage in deep collaborations (Muthusamy & White, 2005). For some of the European university alliances, already existing trust-based relationships might have formed the foundation for establishing the new alliance. For others, trust will have to be built as the alliance is formed. As such, trust is a distinguishing feature that might create strong ties between organizations and allow for new knowledge to be created through what Vedres and Stark (2010: 1183) label inter-cohesion—where groups of organizations “fold into” each other in ways that enable persistence over time. While trust can be said to be a basic condition for such persistence (Maassen & Stensaker, 2022), research on inter-organizational relations has demonstrated that there are a number of potential factors that can influence the life-span of an alliance, including the ways knowledge within the alliance is shared, the type of knowledge the alliance possesses, alliance characteristics and cultural factors (Inkpen & Tsang, 2007; van Wijk et al., 2008).

### Key dimensions impacting university alliances

The meta-organizational framework offers rich possibilities for studying the EUI. In this section, we introduce four dimensions (coordination, conflict resolution, commitment and cultural characteristics) which—from instrumental or institutional perspectives—are assumed to affect key features of university alliances. All four dimensions—and the ways they are mixed—may contribute to the creation of specific logics within an alliance (Thornton et al., 2012), which may lead to the institutionalization of taken for granted practices and ways of doing things that may transform an alliance into a persistent and long-lasting entity (Selznick, 1957; Boltanski & Thèvenot, 1991).

The degree of organizational *coordination* provides information on the potential a given university alliance may have for consistent performance over time. Organizational coordination can take place in various ways, from developing more loose internal networks to the establishment of a formal organization (Owen-Smith & Powell, 2008; Torfing, 2012). Coordination can also take place through the development of standards and rules, which over time become accepted as guidelines for organizational action (Brunsson & Jacobsson, 2000). To establish various types of internal coordination mechanisms may in this respect signal an interest in aligning the different governance traditions of the member universities (Ahrne & Brunsson, 2008). Characteristics of the persons selected to handle coordination issues are also of importance (Hustedt & Danken, 2017). While professional administrators are carriers of in-depth expertise in specific areas, they regularly struggle with seeing problems from a more holistic perspective. The academic leadership, on the other hand, might have more holistic perspectives, but lack in general the in-depth expertise necessary to find practical solutions (Hustedt & Danken, 2017).

University alliances also need mechanisms for *conflict resolution*. As members may have divergent interests and different preferences on a number of issues, developing an agreed upon practice for solving conflicts can be seen as a mechanism for securing the persistence of an alliance. The relatively weak central authority found in most meta-organizations may pave way for two types of conflict resolution: voting or consensus-oriented practices (Ahrne & Brunsson, 2005: 441). While voting mechanisms may allow quick solutions, this is also a mechanism that may have a potential negative impact on the alliance, especially if some members always find themselves on the losing side. Consensus-oriented conflict mechanisms may be quite slow, but they have at the same time the potential of strengthening the cultural bonds and trust between the members (Vedres & Stark, 2010).

Whether university alliances are ultimately persistent is also dependent on the *commitment* of their members (Ahrne & Brunsson, 2008). As many of the selected European university alliances have been set up after the EUI call by the EC, there is a risk that the commitment to engage in the alliance will be reduced if the EC funding stops (Beerkens, 2004), or if an attractive alternative to the current European university alliance emerges (Ahrne & Brunsson, 2005). The rationale and engagement these university alliances have is thus an important signifier for future persistence (Beagles, 2022). Justifications that match the identity of the members could be an indication of more long-term commitment (Boltanski & Thèvenot, 1991). At the same time, a number of the alliances may have been constituted by universities that were collaborating already before they were selected by the EUI programme. Therefore, the commitment of these alliances to continue their joint engagement might not depend primarily on the availability of EC funding.

*Cultural characteristics* may also affect the persistence of university alliances (Muthusamy & White, 2005). Such characteristics are related to whether the members of an alliance share similar norms and values, have similar historical trajectories and find an effective balance between integrating into in a new alliance and preserving their historical institutional identity (Labianca et al., 2001). If new practices and processes in an alliance deviate too much from existing ways of doing things, individual members may find it easier to withdraw from the alliance or to be less engaged in joint activities (van Wijk et al., 2008).

The four dimensions identified should not be seen as mutually exclusive. Studies have shown that factors related to culture and identity may have implications for the governance arrangements established (Beagles, 2022), and that formal governance designs indeed affect the cultural practices that are developed over time (Hustedt & Danken, 2017).

## Data and methods

The data used in the study presented in this article stems mainly from interviews with actors having a leadership position in a European university alliance, such as general alliance secretary or coordinator, and chairperson/head of the management or executive alliance board. The ten interviewees are numbered (R1–R10). For anonymity reasons, these numbers are not identical to the listing of alliances in Table 1. Interview requests were sent to all 41 alliances, and ten alliances responded positively (almost 25% of the existing alliances). The interview guide was semi-structured and was sent to the interviewees before the interview. The interviews covered six predefined areas of interest: governance structures (bodies, budget management and mission), comparison between being in an alliance and participating in other EU projects, main educational programmes and activities, research activities, transmission of European values and principles and future developments. This article presents the main findings with respect to governance structures and related themes, such as decision-making bodies and mission, vision and identity. The findings with respect to the other five areas will be published separately.

**Table 1:** Basic features of selected European alliances

Alliances	Number of member institutions	Number of students	Number of staff
Alliance 1	6	115,000	26,000
Alliance 2	6	160,000	13,000
Alliance 3	7	104,000	8500
Alliance 4	9	71,000	8100
Alliance 5	9	305,000	57,000
Alliance 6	9	313,000	45,000
Alliance 7	9	334,000	44,000
Alliance 8	9	474,000	64,000
Alliance 9	11	> 500,000	> 100,000
Alliance 10	13	222,000	35,000

Source: alliance websites and factsheets

Due to Covid-19 restrictions, interviews were conducted virtually by one of the researchers, and lasted between 35 and 45 min. All interviews were recorded and transcribed. Supplementary data were collected from the webpages and relevant documents of the selected alliances—including organizational charts, overview of working groups, task forces and other information of relevance for shedding light on the organization and governance of the alliance. Of the ten alliances included in this study, four belong to the first call and six to the second, representing 86 European higher education institutions^2^ (summer 2022). These ten alliances span 25 countries, ranging from 12 member institutions from France participating in these alliances to six countries with one institution being a member of one of the alliances.

In Table 1, some basic features of the selected alliances are presented. As can be seen in the table, there is a significant variety in student and staff numbers among the selected alliances, while the largest alliance has more than double the number of member institutions (13) as the two smallest (6).

The interview data were thematically analysed using the four conceptual dimensions introduced above. The first round of analysis was done by one of the researchers that did not conduct the interviews. In this round, codes related to each dimension were used to extract samples of data from the interviews. The extracted samples were in the second step checked for reliability by the two other researchers, and all three researchers agreed on the data selected in the final step. One of the researchers has a central position in a European university alliance. This researcher did not participate in the interviews, but was part of the interpretative process afterwards. While such involvement may represent an interpretative bias, it could also have advantages with respect to an insider understanding of the issues at stake. The position of this researcher was taken into account in the joint analyses undertaken.

## Results

### Coordination

Data from the websites of the selected university alliances indicates that all have developed, or are in the process of developing a range of different coordination mechanisms for aligning organizational practices in a range of areas. These organizational coordination mechanisms are, not surprisingly, closely related to the initial logic behind the establishment of university alliances—mobility schemes, quality assurance and sharing of data.

In general, the coordination mechanisms established in the educational area can be characterized as standards and generic guidelines rather than strict rules and regulations. In this sense, the coordination mechanisms are more on the softer side although they are seen as crucial for the internal functioning of the alliances. As one of our interviewees (R2) argued:

*All the things we do are known problems like seamless mobility, trying to get rid of different obstacles between different countries, open up courses to our partners, forming a joint course catalogue. All these things are not very innovative but they form a very important basis to get our student community together*

Three of the university alliances have also formalized their alliance within a legal framework making it a self-standing legal entity. Such formalizations tend to be accompanied by other forms of organizational coordination in the form of a secretariat and a general secretary as one of our interviewees (R8) explained:

*Formally, our alliance is a Belgian non-profit association (AISBL) with a separate secretariat and a general secretary*

That some alliances are forming separate legal entities is a development in line with the new European Strategy for higher education (European Commission, 2022), even though individual members of an alliance may want to protect their own institutional autonomy, and want to avoid being bounded by legal frameworks outside their own country. Using the AISBL as an example, this legal framework is less constrained by legal rules, which implies that those setting it up have considerable room for discretion with respect to the configuration of the entity, the membership rights and their obligations. Nonetheless, establishing an AISBL implies some formal organizing with general assemblies, board of directors etc.

Another coordinating mechanism used by some of the selected alliances is related to how the missions and academic profile of an alliance match those of its members. This is not least visible through aligned strategies, but also through the personnel overlap in the management of the alliances and the member universities. The representation of senior leadership is a distinct characteristic of the alliances with presidents/rectors and vice-presidents/vice-rectors occupying key roles. As one of our interviewees (R6) indicated, the result is that:

*(alliance) XX is really embedded in the organizational structures of each university, this is more than just a project but involves people at the highest levels*

Our data also suggests that missions and profiles in some respects converge between alliances. Comparisons of missions and academic profiles indicate that many alliances emphasize activities related to climate, environment, SDG objectives, democracy etc. Many of the associated partners in the alliances from outside higher education also have a profile in these areas, although it seems that these associated partners are seldom involved in the internal coordination mechanisms established within the alliances. While it lies beyond the scope of the article to analyse in more detail the nature of the convergence dynamics, it can be argued that the EUI call plays an important role in the initial profiling of the alliances. Future studies should examine whether this initial convergence will become a remaining, institutionalized feature of European university alliances’ mission and profiles, or alternatively, whether alliances will move away from the core profile areas of the EUI call and develop more unique missions and profiles.

### Conflict resolution

Documents available concerning the governance of the university alliances reveal some similarities in formal structures, often in the form of general assemblies (consisting of rectors/presidents) and a management/executive board (often consisting of vice-rectors/vice-presidents) taking decisions regarding alliance activities. While documents are less informative with respect to how decisions actually are made, the interviews with representatives of the selected alliances indicate that consensus-oriented procedures dominate decision-making.

When asked about the key decision-making body of their alliance—regardless of how it is labelled—interviewees are clear that various forms of informal bargaining, dialogue and persuasion are key mechanisms. Sometimes, the key decision-making body also engages in mediation processes at lower levels with the ambition to find pragmatic ways to solve problems:

*And the management board is also the go-to board when there is something where the partners can’t agree, the management board would be the mediator to solve these problems and to mediate between different opinions*. (R10)

At the same time, it is still rare that consensus-oriented decision-making is formalized in statutes and in internal regulations. However, there are also alliances that have made consensus-oriented decision-making into a rule:

*On decision-making in general, our rule of principle is that decisions are made by consensus particularly in the steering committee*. (R4).

Most interviewees underline that agreeing on decisions is seldom a problem, and that most items on the agenda have been agreed upon at lower levels in the alliance—in the different work packages or within specific action lines or task forces. However, also at lower levels in the alliances, the consensus orientation tends to prevail as illustrated by the following quote from one of our interviewees (R3):

… *the whole governance is very trust based and very consensual, because in every work package you will always find representatives from everybody, from every institution, and even if there is a chair…it`s always a collective outcome of this work package that is collectively relegated on the upper level*

As this quote illustrates, it seems that collective organizing at work package level and within action lines triggers more collegial than hierarchical decision-making. Formal leadership and management positions, such as being a coordinator or work package leader, seem to take on more of an administrative than a managerial role.

### Commitment

It is not surprising that formal documents associated with alliance formation express a high level of commitment from the members. As many of these documents were also used as part of the application to receive funding, it can be expected that lofty ambitions and a language intended to fit the funder’s preferences dominate.

Nonetheless, the interviewees are still very convincing in their re-affirmation of the commitments to the alliances, and provide various examples of how such commitment comes to the fore—for example, with respect to the number of people from the universities that are engaged in alliance processes, the time spent on alliance issues or own resources spent beyond the support received from the EU.

Several of the interviewees also highlighted the individual commitment that key persons involved in the alliance formation are showing, which may create a spillover effect on the persistence of the alliances. Many in senior leadership positions within the universities that make up the alliances were also central in developing and shaping the initial proposal, creating what seems to be a personal commitment to the alliance and its development. These key persons have continued to be engaged in the alliance also after its establishment:

*In the proposal phase the vice-presidents, now the members who sit on the management board, were already very much involved in shaping the proposal. It was clear that that would be the core management team later*. (R9)

Some of the interviewees underline that the formal establishment of the alliance as an international non-profit foundation also is an example of a high level of commitment beyond the specific project funded through Erasmus + . Establishing a formal foundation for a project that initially only is funded for 3 years by the EC can be seen as premature and a high-risk decision. However, the majority of our interviewees underline that the 3-year pilot project is subordinated to the longer-term vision embedded in the alliances, exemplified by the following quote:

*For us, XX is first of all a commitment of our universities that of course is supported strongly by the Erasmus + project, but in any case we want to make sure that it remains even beyond the European funding*. (R6)

It is also worth noticing that a few alliances even have ambitions of developing beyond being in a strategic partnership with other universities—hinting about how they want to become a formally integrated institution in a long-term perspective. While such ambitions are rare, they do signal a very high level of commitment.

### Cultural characteristics

Analyses of membership and academic profiles of the 41 European university alliances reveal that the large majority of the alliances consist of like-minded universities—for example, having a profile as research-intensive comprehensive universities, technical universities, regional universities or innovative universities.

Being like-minded seems also to have paved the way for forming the alliance in the first place. Previous collaborations and knowledge of each other are frequently reported among our interviewees as being a key factor when identifying partners to join up with:

..*we have already known each other for decades, so that there is a lot of common trust, if you have to work on-line of course it`s not nice, but it really helps that we know each other and trust each other*. (R2)

As emphasized in the quote, previous knowledge and experience in working together have created a high level of trust among partners in the alliance. Disciplinary networks being set up in specific areas within the alliances are also enhancing cultural convergence.

However, cultural characteristics within the alliances are not always aligned. For example, initiatives opening up for more inter-disciplinary collaboration, where different disciplinary logics create tensions that have to be solved, are reported to be more time-consuming to organize. It is also important to remember that many European university alliances have associated partners—local municipalities, external research institutes, non-profit public and private organizations etc.—that may have profiles and missions that are very different from the ones emphasized by the universities in the specific alliance. Interviewees report that some of the biggest challenges of the alliances are related to find effective ways to engage their associated partners, as reported by one of our interviewees (R5):

…* the most difficult for us is to have real involvement by our associated partners because you know there should be mutual benefit, so since we have very different partners with very different missions…so we have local authorities, regional councils and so on and it is very difficult to find common ground to develop interest and commitment from the different partners.*

Finding common ground is not least a matter of matching cultural characteristics where interviewees highlight the challenges of finding appropriate ways of working together, prioritizing tasks and agreeing on what outcomes are preferable.

## Discussion

While the EUI programme is still unfolding, our study provides pointers as to the persistence of the alliances selected by the programme, that is, whether the European university alliance as a new (meta-)organizational form has the potential to become institutionalized in the European Higher Education Area (EHEA). Returning to our four dimensions examined, they provide indications of long-term persistence although with some exceptions.

The coordination mechanisms established range from those intended to standardize core operations within the alliances (mobility schemes and quality assurance) to mechanisms that formalize the alliances as meta-organizations (setting it up as an independent association/foundation). The extent of coordination mechanisms established suggests that the alliances are actively attempting to modify one of the main problems of meta-organizations, their vulnerable starting point regarding the development of effective internal governance (Ahrne & Brunsson, 2008).

However, it cannot be taken for granted that all coordination mechanisms established are pulling the alliances in the same direction (Vukasovic & Stensaker, 2018). As the principle of consensus seems to be established in the majority of alliances at all decision-making levels (from work packages to management boards), a number of (micro-)decisions are made at various levels running the risk of being disconnected from overarching aims and objectives.

In addition, the organization of the day-to-day work, for example, in the form of work packages, may result in internal silos reducing the potential for knowledge transfer and organizational learning (Hustedt & Danken, 2017; Muthusamy & White, 2005; van Wijk et al, 2008). A number of the alliances in our study recognized this risk. They try to counter it by including all partners in every alliance activity, and by sometimes appointing task forces that work on problems cutting across different work packages.

The strong consensus orientation in decision-making within the alliances will also reduce, in principle, internal tensions and interest differentiation among alliance members (Ahrne & Brunsson, 2005; Hustedt & Danken, 2017). The main challenge with this form of conflict resolution is the time aspect—the fact that decisions may take some time to agree upon runs the risk of missing opportunities and failing to achieve what have been set out as objectives for the projects within the deadlines set, not least toward the EC. At the same time, consensus-oriented conflict resolution may enhance the commitment from members and bring them closer together as partners (Beagles, 2022; Vedres & Stark, 2010)—hinting that European university alliances may have a life beyond the initial economic support from the EC.

Nevertheless, the interviews suggest that the reported commitment of partners within the alliances is strong regardless of the possible conflict mechanisms in place. Pre-existing knowledge of and working relationships with partners before alliance establishment are important factors explaining this commitment. It could be argued that activities related to the establishment of the EHEA have created interactions and relationships (Vukasovic, 2017) that paved the way for the European university alliances. From this perspective, the establishment of university alliances is not such a big step forward as core activities—student mobility and collaboration on educational offerings—have been central for European university cooperation for several decades. The new practices established through alliance formations may in this respect be seen as natural steps forward from previous forms of cooperation, and an innovation that is not that dramatic and revolutionary (Lounsbury & Crumley, 2007). Individual universities may interpret their alliance membership as non-threatening with respect to their identity and history (Labianca et al., 2001). However, even though the core academic activities are the same, there is a difference between the ways in which they were organized before (as projects to implement) and the new ways of organizing them within the alliances. As has been argued in most of the interviews, the European university alliances are not just a project. They represent something more complex, with different layers, challenges and systems of governance and management.

The fact that many alliances seem to consist of rather like-minded institutions is also a factor that may enforce the long-term persistence of the alliances established. Similar institutions and academic profiles make it easier to collaborate as social and cultural practices are more aligned in the first place (Beagles, 2022; Inkpen & Tsang, 2007). However, current alliances might have one particular feature that can be challenging with respect to culture and identity—the number of associated partners from outside higher education. Alliances already report that these partners are challenging to engage and integrate in alliance activities and that they operate with different logics than the member universities. The challenge is then to create sense out of dissonance regarding these partners (Stark, 2009)—and find ways to develop the alliances in transformative ways (Stensaker, 2018). Based on our data, it seems that these associated partners are only partially integrated in the coordination mechanisms established within the alliances. While integrating them more structurally may seem sensible, such involvement in established coordination mechanisms may also put strains on existing features that may enhance alliances’ integration (consensus orientation, commitment and cultural characteristics).

## Closing reflections

The development of European university alliances can have many different implications for the EHEA. In this article, the focus has been on the potential persistence of the selected alliances where we have identified factors that may reinforce the institutionalization of university alliances. At the same time, we have also noticed factors that may de-stabilize the established partnerships.

Our meta-organizational theoretical conceptualizations could be applied in several ways for future examinations of alliance roles and effects. For example, more in-depth analysis of the cultural characteristics and identity of the new alliances could shed light on how the landscape of higher education in Europe is affected by the EUI. In an earlier study, Vukasovic and Stensaker (2018) found that various university interest organizations and alliances tend to expand their agenda and scope over time. Will the same development take place within the EUI—perhaps implying that research and innovation activities might become more important for alliances?

University alliances could potentially also change the landscape in other ways. Ahrne and Brunsson (2005) have argued that meta-organizations have some built-in logics that may lead to new and larger strategic partnerships as similar types of alliances may see advantages in collaboration. The fact that the EC currently is signalling more economic support for larger alliances could provide a strong incentive for inter-alliance mergers. The potential emergence of large alliances of universities might also have implications with respect to power and authority within the (EHEA), not least as regards the impact of new influential actors and for the policy-making process in general (Chou & Gornitzka, 2014; Vukasovic et al., 2018).

The internal governance of university alliances is also worth pursuing in future studies. How university alliances are able to organize and utilize their work and activities in ways that facilitate knowledge transmission, learning and innovation within the partnership is here of particular interest, as learning and innovation are key reasons why many universities decide to enter alliances. Internal governance is a key factor in this respect as it sets the boundaries and defines the opportunities for organizational dynamics within the alliances (Inkpen & Tsang, 2007; van Wijk et al., 2008). Meta-organizations may be ineffective in the short run, but depending to a large extent on the effectiveness of their internal governance, they tend to gain importance over time (Ahrne & Brunsson, 2005: 447).

## Footnotes

1 The term “university” refers to all types of higher education institutions in the Strategy (European Commission, 2022: 1).

2 This figure refers to the total number of institutions that were a formal member of one of the 10 included European university alliances summer 2022. A number of these alliances had welcomed new members since their original application to the EUI programme.

## References

[CR1] Ahrne G, Brunsson N (2005). Organizations and meta-organizations. Scandinavian Journal of Management..

[CR2] Ahrne G, Brunsson N (2008). Meta-organizations.

[CR3] Beagles, J. (2022). Institutional logics and the multiorganizational governance arrangements of humanitarian INGOs. *Nonprofit Management and Leadership*, 1-25.10.1002/nml.21507

[CR4] Beerkens H (2004). Global opportunities and institutional embeddedness: Higher education consortia in Europe and Southeast Asia.

[CR5] Bleiklie I, Enders J, Lepori B (2017). Managing universities. Policy and organizational change from a Western European comparative perspective.

[CR6] Boltanski L, Thévenot L (1991). On justification: The economies of worth.

[CR7] Brunsson N, Jacobsson B (2000). A world of standards.

[CR8] Capano G, Pritoni A, Vicentini G (2020). Do policy instruments matter? Governments’ choice of policy mix and the higher education performance in Western Europe. Journal of Public Policy.

[CR9] Chou M-H, Gornitzka Å (2014). Building the knowledge economy in Europe: New Constellations in European research and higher education governance.

[CR10] Djelic M-L, Sahlin-Andersson K (2006). Transnational governance: Institutional dynamics of regulation.

[CR11] European Commission (2020a). *Achieving a European Education Area by 2025 and resetting education and training for the digital age*. Press release 30 September 2020a. Brussels: European Commission. Accessed 21 January 2022: https://ec.europa.eu/commission/presscorner/detail/en/IP_20_1743

[CR12] European Commission (2020b). *On achieving the European Education Area by 2025*. COM(2020b) 625 final. Brussels: European Commission. Accessed 21 January 2022: https://ec.europa.eu/education/sites/default/files/document-library-docs/eea-communication-sept2020b_en.pdf

[CR13] European Commission (2022). *On a European Strategy for universities*. COM(2022) 16 final. Brussels: European Commission. Accessed 15 February 2022: https://eur-lex.europa.eu/legal-content/EN/TXT/PDF/?uri=CELEX:52022DC0016

[CR14] Fukuyama F (2015). Political order and political decay. From the industrial revolution to the globalisation of democracy.

[CR15] Gornitzka Å, Maassen P, de Boer H (2017). Change in university governance structures in continental Europe. Higher Education Quarterly.

[CR16] Greenwood R, Raynard M, Kodeih F, Micelotta ER, Lounsbury M (2011). Institutional complexity and organizational responses. The Academy of Management Annals.

[CR17] Hustedt T, Danken T (2017). Institutional logics in inter-departmental coordination: Why actors agree on a joint policy output. Public Administration.

[CR18] Inkpen A, Tsang E (2007). Learning and strategic alliances. The Academy of Management Annals.

[CR19] Jungblut, J., Maassen, P. & and Elken, M. (2020). Quo Vadis EHEA: Balancing structural continuation and political variety. In Curaj, A. et al. (Eds.), *European higher education area: Challenges for a new decade* (pp. 391–416). Springer. 10.1007/978-3-030-56316-5_25

[CR20] Labianca G, Fairbank JF, Thomas JB, Gioia D (2001). Emulation in academia: Balancing structure and identity. Organization Science.

[CR21] Lounsbury M, Crumley E (2007). New practice creation: An institutional perspective on innovation. Organization Studies.

[CR22] Maassen P, Olsen JP (2007). University dynamics and European integration.

[CR23] Maassen P (2017). The university’s governance paradox. Higher Education Quarterly.

[CR24] Maassen P, Stensaker B (2011). The knowledge triangle: European higher education policy logics and policy implications. Higher Education.

[CR25] Maassen, P. & Stensaker, B. (2022). Trust and higher education governance in Norway and the United Kingdom. In Gibbs, P. & Maassen, P. (Eds.), *Trusting in Higher Education. A multifaceted discussion of trust in and for higher education in Norway and the United Kingdom* (pp. 17-37). Springer.

[CR26] Muthusamy S, White MA (2005). Learning and knowledge transfer in strategic alliances: A social exchange view. Organizational Studies.

[CR27] Owen-Smith J, Powell WW, Greenwood R, Oliver C, Sahlin K, Suddaby R (2008). Networks and institutions. The Sage handbook of organizational institutionalism.

[CR28] Sakamoto R, Chapman D (2011). Cross-border partnerships in higher education.

[CR29] Scott WR (2014). Institutions and organizations: Ideas, interests and identities.

[CR30] Selznick P (1957). Leadership in administration.

[CR31] Shattock M (2014). International trends in university governance.

[CR32] Stark D (2009). The sense of dissonance: Accounts of worth in economic life.

[CR33] Stensaker B (2018). University alliances: Enhancing control, capacity, and creativity in dynamic environments. Educational Studies Moscow.

[CR34] Thornton P, Ocasio W, Lounsbury M (2012). The institutional logic perspective: A new approach to culture, structure and process.

[CR35] Torfing, J. (2012). Governance networks. In D. Levi-Faur (Ed.), *The Oxford handbook of governance*. Oxford University Press.

[CR36] van Wijk R, Jansen JJ, Lyles MA (2008). Inter- and intra-organizational knowledge transfer: A meta-analytical review and assessment of its antecedents and consequences. Journal of Management Studies.

[CR37] Vedres B, Stark D (2010). Structural folds: Generative disruption in overlapping groups. American Journal of Sociology.

[CR38] Vukasovic M (2017). Stakeholder organizations in the European higher education area: Exploring transnational policy dynamic. Policy and Society.

[CR39] Vukasovic M, Stensaker B (2018). University alliances in the Europe of knowledge: Positions, agendas and practices in policy processes. European Educational Research Journal.

[CR40] Vukasovic, M., Jungblut, J., Chou, M-H., Elken, M. & Ravinet, P. (2018). Multi-level, multi-actor and multi-issue dimensions of governance of the European higher education area and beyond. In Curaj, A. et al. (Eds.) *European Higher Education Area: The impact of past and future policies.*

